# Redo bilateral lung transplantation in a previous heart-lung transplant recipient for invasive pulmonary scedosporiosis

**DOI:** 10.1016/j.jhlto.2025.100247

**Published:** 2025-04-01

**Authors:** Akshay Chauhan, Nischal Ranganath, John P. Scott, Paschalis Vergidis, Anja C. Roden, Philip J. Spencer, Mauricio A. Villavicencio, Richard C. Daly, Sahar A. Saddoughi

**Affiliations:** a Department of Cardiovascular Surgery, Mayo Clinic, Rochester, Minnesota; b Section of Infectious Disease, Department of Medicine, Mayo Clinic, Rochester, Minnesota; c Division of Pulmonary, Department of Medicine, Mayo Clinic, Rochester, Minnesota; d Department of Laboratory Medicine and Pathology, Mayo Clinic, Rochester, Minnesota; e Division of Thoracic Surgery, Department of Surgery, Mayo Clinic, Rochester, Minnesota

**Keywords:** CLAD, heart-lung transplant, olorofim, redo-lung transplant, *Scedosporium* pulmonary infection

## Abstract

Redo-bilateral lung transplant in previous heart-lung transplant is a rare operation. We describe our surgical and medical management experience with a heart-lung transplant recipient who developed chronic lung allograft dysfunction and invasive *Scedosporium* apiospermum infection. The patient underwent a redo-bilateral lung transplant followed by a combination of inhaled voriconazole, caspofungin, and olorofim for a prolonged period. We observed no issues with bronchial anastomosis healing, and the patient is doing well on 1-year follow-up. This report outlines a successful management approach to this rare complication of heart-lung transplant recipients.

## Background

Redo-lung transplants in patients with prior heart-lung transplants are rare but are expected to become more frequent due to rising numbers of combined procedures. We report a case of a heart-lung transplant recipient with chronic lung allograft dysfunction (CLAD) and severe, treatment-resistant pulmonary scedosporiosis necessitating redo-bilateral lung transplant. This represents the first documented case of refractory *Scedosporium* apiospermum infection in such recipients treated with this approach,^1^ underscoring both technical challenges and pharmacological advancements in post-transplant infection management.

## Case report

**Figure 1:**
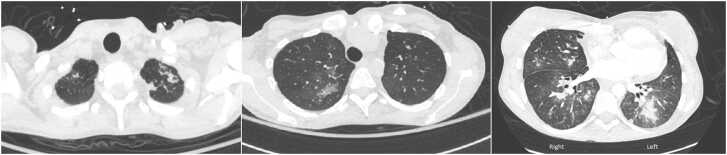
Preoperative computed tomography scan of the chest with contrast demonstrating consolidative nodular opacity in the left lower lung with scattered opacities throughout the remaining lungs, with bronchial and lung parenchyma changes suggestive of CLAD. CLAD, chronic lung allograft dysfunction.

The patient is a 35-year-old woman who underwent combined heart-lung transplant 7 years ago presented with stage 3 CLAD and progressive respiratory failure. Her sputum culture had growth of *Scedosporium apiospermum*, for which she received posaconazole and caspofungin. She had failed prior therapy with voriconazole, and olorofim was not available before transplant. Despite antimicrobial susceptibility–directed therapy, the patient continued to have progression of chronic respiratory failure with persistent isolation of *Scedosporium* spp. in lower respiratory cultures. Serum galactomannan was negative and 1-3-B-D-glucan was positive (91 pg/ml). Computed tomography of the chest showed features of CLAD and right lower lobe consolidation (Figure 1). Her cardiac allograft function was preserved. Given the concern for poorly controlled invasive fungal infection and CLAD-BOS (bronchiolitis obliterans syndrome) type, the decision was made to proceed with redo-lung transplant. Certainly, the decision to proceed with transplant was not taken lightly, given the surgical complexity of the case in combination with the difficult infection that we knew had seeded the airway.

**Figure 2:**
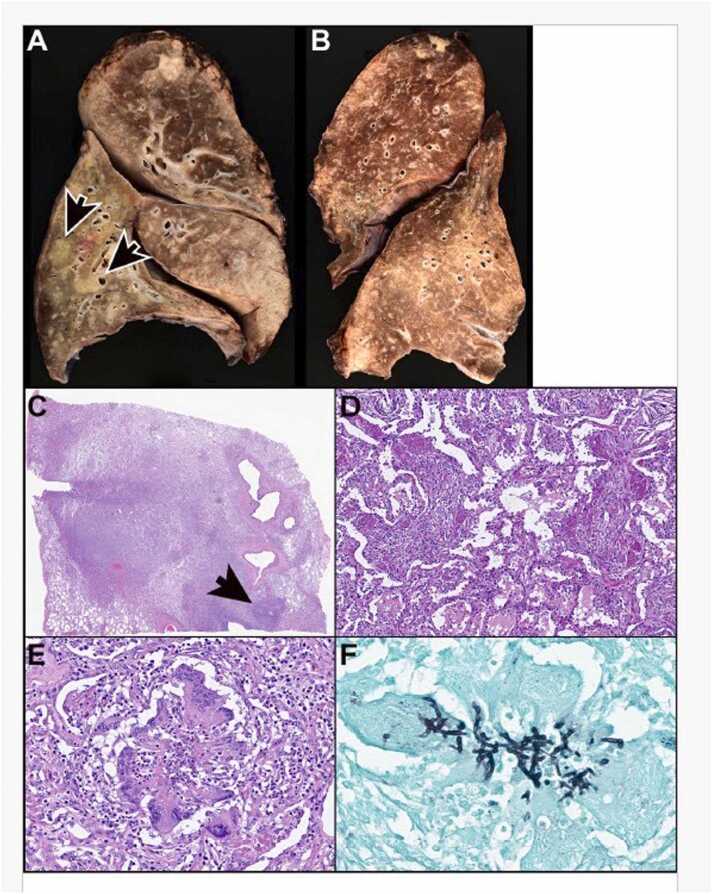
Explanted allograft lungs, International Society for Heart and Lung Transplantation (ISHLT) grade^2^ AXBXC1D1 and obstructive pneumonia. (A) While scattered peribronchiolar (A) and perivascular (B) chronic inflammation is suggestive of B1R and A2 rejection, respectively, those findings can also be seen in infectious processes; therefore, the explanted lungs are graded as AXBX. Also note multinucleated giant cells with cholesterol clefts (arrow A) that are suggestive of obstruction. (C) A bronchiole (arrow) is occluded by young fibrosis (note, fibroblasts occluding the airway lumen) consistent with obliterative bronchiolitis (grade C1 rejection). (D) A Verhoeff Van Gieson (VVG) stain highlights the single elastic lamina of the bronchiole (arrow). (E) This small airway is focally completely occluded by collagen fibrosis (also indicating grade C1 rejection). (F) A single elastic lamina outlines the occluded part of the bronchiole (arrow). Note, a pulmonary artery (arrowhead C) exhibits eccentric intimal fibrosis consistent with grade D1 rejection. This is better visualized using a VVG stain (D) which highlights the external and internal elastic lamina of the pulmonary artery. Magnification, hematoxylin & eosin (H&E) ×10 (A), ×20 (B, C), VVG ×4 (D), ×4 (E), VVG ×4 (F).

The patient underwent a bilateral lung transplant via a transverse thoraco-sternotomy (clamshell) incision. The prior heart-lung transplant had been performed through a midline sternotomy, resulting in dense adhesions that were meticulously dissected and removed. Hilar dissection and the remaining transplant procedure were supported by veno-arterial extracorporeal membrane oxygenation (ECMO). The ascending aorta was cannulated with an 18 Fr fem-flex cannula, while venous drainage was achieved using a 21 Fr multistage venous cannula placed in the right atrium via the right common femoral vein. Hilar dissection, pneumonectomy, and anastomoses of the pulmonary artery and vein cuffs were performed as standard. Donor-to-donor bronchial anastomosis was completed with a 4-0 SH PDS suture, using continuous running sutures on the membranous portion and interrupted sutures on the cartilaginous section. Donor lung ischemia time was under 6 hours. Postoperatively, the patient required a tracheostomy but was successfully weaned from the ventilator by day 14. Patient was managed with triple immunosuppressive therapy—tacrolimus, mycophenolate, and prednisone—along with additional perioperative steroids. Tacrolimus trough levels were maintained within the target range of 6 to 10 mg/ml. The lower target for tacrolimus reflects the patient's long-term use of the drug and the presence of *Scedosporium* infection. Histopathology of the explanted lungs revealed severe tissue-invasive fungal pneumonia, obliterative bronchiolitis, and intimal fibrosis in scattered pulmonary arteries (Figure 2). Operative cultures grew *Scedosporium* spp. complex with diagnosis consistent with invasive pulmonary scedosporiosis.

**Figure 3:**
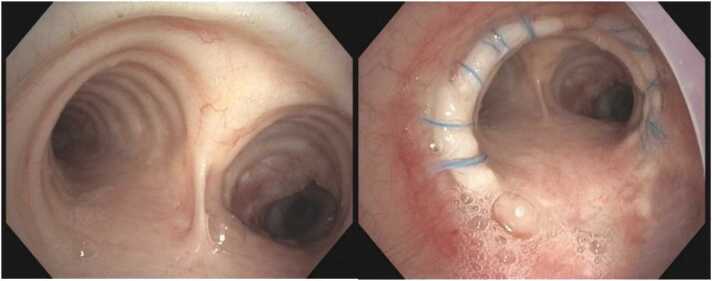
Post redo-lung transplant bronchoscopy images at 2 months follow-up, demonstrating healed suture line and healthy donor bronchial mucosa.

There were no airway complications post surgery (Figure 3). Persistent isolation of *Scedosporium* from respiratory secretions post-transplant and histopathological confirmation of invasive disease in the explanted lungs prompted a treatment regimen comprising olorofim, inhaled voriconazole, and caspofungin during hospitalization of 1 month. This salvage regimen was selected based upon susceptibility testing (Table 1), as well as guideline recommendations supporting voriconazole as agent of choice for scedosporiosis. Upon discharge, she continued olorofim combined with aerosolized liposomal amphotericin B. By 3 months post-transplant, she transitioned to olorofim monotherapy. Bronchoalveolar lavage cultures initially grew *Scedosporium* for up to 6 weeks after olorofim initiation, subsequently yielding negative results. We observed no further microbiologic relapse, continued improvement in respiratory status and the patient continues to do well at 1-year follow-up.

**Table 1:** Antimicrobial Susceptibility Profile of *Scedosporium apiospermum*

Antifungal agents tested	Minimum Inhibitory Concentration (MIC)(mcg/ml)	Breakpoints
Micafungin	0.03	a
Caspofungin	≤0.015	a
Terbinafine	>2	a
Posaconazole	1	a
Voriconazole	1	a
Isavuconazole	8	a
Amphotericin B	2	a
Fosmanogepix	≤0.008	a
Ibrexafungerp	2	a
Olorofim	0.25	a

a No established breakpoints available.

## Discussion

Surgically, the concern in a redo-lung transplant after heart-lung transplant is the vascularity of the recipient's bronchial stump. After a heart-lung transplant in the immediate postoperative period, retrograde perfusion via the pulmonary circulation supports the blood supply to the donor bronchus. Over 1 to 2 months, collaterals arising from coronary circulation supply the donor carina and the proximal bronchus, and donor-to-donor bronchial anastomosis in such patients heals appropriately without complications.^3^ The redo-lung transplant was performed 7 years after the first transplant and the bronchus on the recipient's side was well collateralized. We did not encounter any airway complications in the postoperative period.

Medically, *Scedosporium* pulmonary infection poses significant treatment challenges owing to high-level resistance to common antifungals, with voriconazole and posaconazole considered first-line therapy.^4^ Despite improved survival with adjunctive surgery and treatment with voriconazole, mortality from invasive scedosporiosis remains high (up to 58%).^5^ A recent multinational survey demonstrated that pretransplant *Scedosporium* isolation was considered an absolute contraindication to lung transplant in 10% of centers.^6^ Fortunately, novel antifungal agents, including olorofim and fosmanogepix, have recently emerged as antimicrobials with in vitro and in vivo activity against rare molds, including *Scedosporium* and *Lomentospora* spp. Olorofim acts through reversible inhibition of the catalytic activity of dihydroorotate dehydrogenase, thereby preventing de novopyrimidine biosynthesis necessary for fungal cell wall synthesis.^7^ As noted in this case of treatment-refractory pulmonary *Scedosporium* infection, the use of olorofim as a combination antifungal regimen in the setting of redo-lung transplant may provide a novel salvage strategy for infection.

## CRediT authorship contribution statement

**Akshay Chauhan:** Conceptualization, Methodology, Writing – original draft, Visualization. **Nischal Ranganath:** Writing – review & editing, **John P. Scott:** Writing – review & editing. **Paschalis Vergidis:** Writing – review & editing. **Anja C. Roden:** Writing – review & editing. **Philip J. Spencer:** Writing – review & editing. **Mauricio A. Villavicencio:** Writing – review & editing. Richard C. Daly: Writing – review & editing. **Sahar A. Saddoughi:** Supervision, Conceptualization, Methodology, Writing – review & editing, Project administration.

## Disclosure statement

The authors declare that they have no known competing financial interests or personal relationships that could have appeared to influence the work reported in this paper.

We acknowledge the lung and heart transplant teams who take excellent care of our transplant patients.
